# Assessment of Amikacin- and Capreomycin-Related Adverse Drug Reactions in Patients with Multidrug-Resistant Tuberculosis and Exploring the Role of Genetic Factors

**DOI:** 10.3390/jpm13040599

**Published:** 2023-03-29

**Authors:** Lauma Freimane, Linda Barkāne, Agnija Kivrane, Darja Sadovska, Viktorija Ulanova, Renāte Ranka

**Affiliations:** 1 Faculty of Pharmacy, Riga Stradiņš University, Dzirciema Street 16, LV-1007 Riga, Latvia; 2 Latvian Biomedical Research and Study Centre, Ratsupites Street 1, k-1, LV-1067 Riga, Latvia; 3 Tuberculosis and Lung Diseases Center, Riga East Clinical University Hospital, LV-1038 Riga, Latvia

**Keywords:** MDR-TB, amikacin, capreomycin, ototoxicity, nephrotoxicity

## Abstract

Following the introduction of all-oral treatment regimens for patients with drug-resistant tuberculosis (TB), second-line injectable drug applications have been reduced in the last few years. However, they are still important for anti-TB therapy. This study aims to analyze the occurrence of amikacin- and capreomycin-related adverse drug reactions (ADR) in patients with multidrug-resistant tuberculosis (MDR-TB) and evaluate the role of multiple patient-, disease-, and therapy-related factors on the frequency of the observed adverse events. In addition, the possible role of genetic risk factors was studied by full-length mitochondrial DNA sequencing. Toward this aim, we retrospectively evaluated 47 patients with MDR-TB who received amikacin and/or capreomycin. In total, 16 (34.0%) patients developed ototoxicity and 13 (27.7%) developed nephrotoxicity, including 3 (6.4%) patients who experienced both adverse events. Ototoxicity development was more common in patients who received amikacin. No other factors showed a significant impact. Nephrotoxicity was likely associated with previous renal health impairment. Full mitochondrial genome sequencing did not reveal any specific ADR-associated variants, and results showed no differences in adverse event occurrence for any specific variants, mutation count, or mitochondrial haplogroup. The absence of the previously reported ototoxicity-related mtDNA variants in our patients with ototoxicity and nephrotoxicity highlighted the complex nature of the ADR occurrence.

## 1. Introduction

Globally, tuberculosis (TB) remains a public health crisis and the second leading cause of death from an infectious agent (SARS-CoV-19 takes first place) [1]. Multidrug-resistant tuberculosis (MDR-TB) is caused by *Mycobacterium tuberculosis (Mtb*) that are resistant to at least isoniazid and rifampicin, the most effective anti-TB drugs [2]. MDR-TB remains an important public health problem; the Global TB Report showed that the burden of drug-resistant TB in 2021 increased by 3% from 2020, with 450,000 cases of rifampicin-resistant or MDR-TB reported [3]. In the case of MDR-TB, second-line antimicrobials should be used, which often cause unwanted adverse events, and the therapy is also longer. Before 2018, aminoglycosides, such as streptomycin, amikacin, kanamycin, and polypeptide capreomycin, were widely used as second-line injectable agents in MDR-TB treatment. Following the introduction of bedaquiline and all-oral regimens, amikacin and streptomycin have been ranked as Group C agents, whereas the use of kanamycin and capreomycin is no longer recommended [4]. Although second-line injectable drug applications in the last few years have been reduced, they are still important for anti-MDR-TB therapy and may be included in longer regimens.

The use of second-line injectable agents often comes with adverse drug reactions (ADR) that may become the cause of treatment discontinuation. All aminoglycosides have the potential to produce reversible and irreversible vestibular, cochlear, and renal toxicity and neuromuscular blockade [5]. Capreomycin is an antimycobacterial cyclic peptide; however, its antimycobacterial activity and untoward effects are similar to that of aminoglycosides [5]. The molecular mechanism of aminoglycoside-induced toxicity has not yet been resolved; however, it was linked to drug trafficking across endothelial and epithelial barriers, cellular uptake, and disruption of intracellular physiological pathways [6]. The disruption of kidney function tends to be reversible, as damaged and dying proximal tubule cells can be replaced by cellular proliferation [7]. In contrast, ototoxicity can result in irreversible, bilateral, high-frequency hearing loss or vestibular hypofunction [8].

The overall incidence of ototoxicity and nephrotoxicity in patients is difficult to determine, as different studies reported variable results [5]. Patients’ individual susceptibility to aminoglycoside- and capreomycin-induced ADR varies considerably, and several risk factors have been identified including high doses, high plasma concentrations, frequent applications, long treatment periods, renal dysfunction, older age, noise exposure, a pre-existing hearing impairment, diabetes, hypertension, and the co-administration of other ototoxic or nephrotoxic drugs [9,10,11]. In addition, the results of several studies suggested an increased risk of aminoglycoside-associated ototoxicity in patients with mitochondrial mutations, particularly in the 12S rRNA gene [12,13,14]. On the other hand, genetic variants specifically related to capreomycin adverse events have not been reported. 

As an effective host-directed therapy is one of the perspectives to address TB drug resistance challenges, it is thus highly important to apprise factors that may impact the inter-individual variability in ADR occurrence. The aim of this study was to analyze the occurrence of amikacin- and capreomycin-related ADR in patients with MDR-TB in Latvia and to evaluate the role of multiple patient-, disease-, and therapy-related factors. In addition, the possible role of genetic risk factors was studied by full-length mitochondrial DNA (mtDNA) sequencing.

## 2. Materials and Methods

### 2.1. Subjects

In this retrospective study, 47 MDR-TB adult patients admitted to the Centre of Tuberculosis and Lung Diseases, Riga East Clinical University Hospital, were included. The inclusion criteria were: patients with informed consent; over 18 years old; an MDR-TB diagnosis; amikacin or capreomycin injections. No specific exclusion criteria were applied. The patient cohort was collected in the period from the year 2014 to 2017. DNA samples and patient data were obtained in collaboration with the national biobank Genome Database of the Latvian population according to the protocol described in Rovite et al., 2018 [15]. Information on patients with MDR-TB treatment regimens, comorbidities, adverse events, treatment outcome, and doses received was obtained in collaboration with the Centre of Tuberculosis and Lung Diseases. The study was approved by the Central Medical Ethics committee of Latvia (approval No 01-29.1/1).

The treatment regimen for the patients with MDR-TB was chosen based on the *M. tuberculosis* resistance data and following the WHO guidelines of the time [16,17]. The treatment outcome was documented for all but one patient: 38 (80.9%) patients were cured, 7 patients discontinued their treatment, and 1 patient died. Amikacin and capreomycin-related ADR occurrence was analyzed; two adverse event types were observed and thus considered: ototoxicity and nephrotoxicity. Ototoxicity was confirmed if the patient developed clinical sensorineural hearing loss, shown as a hearing impairment in an audiogram, or developed dizziness, vertigo, or tinnitus. Nephrotoxicity was referred to as renal failure due to drug usage. Patient questionnaires were used to identify the following factors: age, sex, smoking experience, daily alcohol consumption, body weight, and height. Body mass index (BMI) was calculated based on height and body weight. 

### 2.2. Full mtDNA Genome Sequencing

Human mitochondrial DNA (mtDNA) sequencing was performed using IonTorrent technology and the Personal Genome Machine (PGM) (Thermo Fisher Scientific, USA). First, the whole mitochondrial genome was amplified via two distinct PCR reactions to yield more than 8 kb long products—A and B fragments—as described previously [18]. A and B amplicons were pooled and cleavaged by sonication into 200–250 bp-long fragments. DNA fragment libraries were prepared using the Ion Plus Fragment Library Kit (Thermo Fisher Scientifc, Carlsbad, CA, USA) and the Ion XpressTM Barcode Adapters Kits (Thermo Fisher Scientifc, Carlsbad, CA, USA). DNA clean-up was performed with NucleoMag^®^ NGS Clean-up and Size Select magnetic beads (MACHEREY-NAGEL GmbH & Co. KG, Dueren, Germany).

The sequencing data were uploaded to the Galaxy web platform, and we used the public server at *usegalaxy.org* to analyze the data [19]. BAM data sets were converted into the FASTQ format using the Convert, Merge, Randomize tool. FASTQ sequences were filtered by quality (the cut-off value = 20; percent of bases in a sequence that must have quality equal to or higher than the cut-off value = 90). The tool Trim Galore! was used to remove adapter sequences from the data file. The tool Bowtie 2 was used to map the trimmed reads to the reference genome. Sequence reads were aligned to the standard revised Cambridge Reference Sequence (rCRS, NC_012920.1, *Homo sapiens* mitochondrion complete genome). Aligned reads were analyzed with the FreeBayes tool for haploid genomes using a population model as shown in Nekrutenko and Ostrovsky, 2021 [20]. The target average read depth of all samples was greater than 15.

Single-nucleotide variants (SNVs) were defined as positions with a nucleotide different from the rCRS; indels were not analyzed. To exclude sequencing errors, each sample was also examined manually using the Integrative Genomics Viewer (IGV) tool [21]. 

VCF files were uploaded to the HaploGrep 2.0 classification tool to determine a detailed mtDNA haplogroup [22]. Mutation analyses were conducted using the HmtVar and MITOMAP databases [23,24].

### 2.3. Statistics

We explored the following patient-, lifestyle- and disease-related factors: age, biological sex (male, female), type of antibiotic used during the therapy (amikacin, capreomycin, or both), number of doses received, smoking status (non-smoker/smoking experience), BMI (underweight/normal/overweight/obese), alcohol consumption (normal/increased), comorbidities (human immunodeficiency virus (HIV), diabetes mellitus (DM), hepatitis C virus (HCV); yes/no), simultaneous antiretroviral (ART) therapy (yes/no), TB incident case (new/recurrent), and we evaluated a possible impact of these variables on the ototoxicity and nephrotoxicity occurrence during the therapy.

By using sequencing data, we assessed the occurrence of ototoxicity and nephrotoxicity in patients with MDR-TB depending on mtDNA genetic variants including mtDNA haplogroups and mutation frequencies in protein, 12S rRNA, and tRNA coding genes. 

Based on the data type, we used the following methods to find out if there was any association between these variables: Chi-square test of independence, Fisher’s exact test, logistic regression, ANOVA, and ANCOVA. XLSTAT and R (4.1.2.) software were used; values of *p* ≤ 0.05 and α = 0.05 were considered significant.

## 3. Results

### 3.1. Otoxicity and Nephrotoxicity Occurrence Rates in Patients with MDR-TB

In our study, 47 patients with MDR-TB received injectable anti-TB drugs. The average age of the patients was 48 years (20–87). Amikacin, capreomycin, or both medications were used in two, twenty-nine, and sixteen patients, respectively (therapy was changed based on the *Mtb* resistance data). On average, during the treatment, patients with MDR-TB received 109 doses (from 6 to 346; SD = 83) of injectable anti-TB agents. The clinical characteristics of the patients are summarized in Table 1. 

The results showed that more than half of the patients with MDR-TB experienced ADR, which could be related to the use of amikacin or capreomycin (26 of 47 patients, 55.3%; Table 1). Ototoxicity occurred in 16 (34.0%) patients, and nephrotoxicity—in 13 (27.7%) patients; among them, three (6.4%) patients experienced both adverse events (Table 1). More specifically, all but one patient with ototoxicity showed clinical hearing loss in high frequencies, and some patients complained of noticeable hearing impairments, which was the reason to discontinue the use of injectables. One patient developed dizziness without a hearing impairment, but four others had dizziness, vertigo, or tinnitus in combination with hearing loss (L. Barkāne, personal communication) [25]. 

### 3.2. Ototoxicity Events Were Not Associated with Treatment Duration

**Figure 1:**
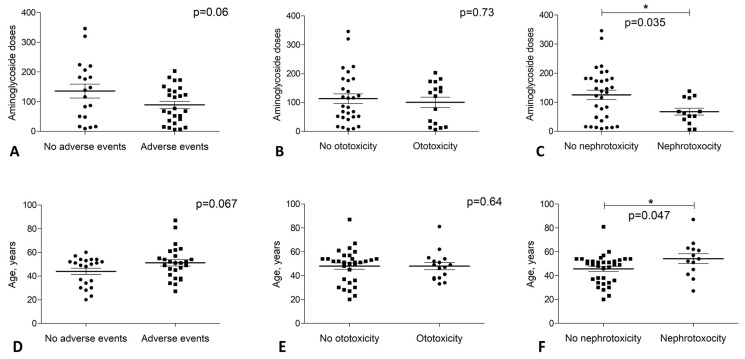
Occurrence of adverse events—ototoxicity and nephrotoxicity—in patients with MDR-TB depending on the age and the number of injections received. (**A**–**C**)—adverse event occurrence depending on injections received. (**D**–**F**)—adverse event occurrence depending on the patient’s age. Mann–Whitney non-parametric test, values of *p* ≤ 0.05 were considered significant. *—*p* < 0.05.

We did not observe a statistically significant association between ADR occurrence and the number of injections received (*p* > 0.05; Figure 1A). Similarly, our data showed that ototoxicity occurrence was not associated with the number of doses received (*p* > 0.05); some patients developed ototoxicity after receiving as little as 6 and 7 injections, whereas patients who received 206 and 346 injections did not experience ADR (Figure 1B).

### 3.3. Nephrotoxicity Events Mostly Occurred at the Beginning of the TB Treatment

The results showed that in our study, patients with nephrotoxicity received significantly fewer injections than patients who did not experience this adverse event (*p* = 0.035; Figure 1C). This finding indicates that nephrotoxicity events mostly occurred at the beginning of the TB treatment.

### 3.4. Nephrotoxicity Events Were Age-Related

It was found that the average age of the patients with nephrotoxicity was significantly greater in comparison to the patients who did not experience this adverse event (*p* = 0.047; Figure 1F). In contrast, age-dependent differences were not observed for the ototoxicity (*p* > 0.05; Figure 1E).

### 3.5. Amikacin Use Was Associated with Ototoxicity Development in Patients with MDR-TB

The association of ADR occurrence with various factors, i.e., BMI, smoking status, alcohol consumption, comorbidities, impaired hearing before treatment initiation, impaired renal function before treatment initiation, the injectable agent used, and recurrence of TB, was analyzed. The results of the univariate analysis revealed that the occurrence of ototoxicity was significantly less frequent in patients receiving capreomycin (*p* = 0.004; Table 1). In accordance, the results of the logistic regression analysis showed that a statistically significant factor for ototoxicity occurrence was the use of amikacin (*p* = 0.049; Chi-Square 3.88; 95% CI: 0.002–0.915) (Supplementary Table S1). The test’s overall specificity reached 90.3%, and sensitivity was 50.0%, with a total of 76.6% correct predictions. 

### 3.6. Factor Analysis for Nephrotoxicity Occurrence

The univariate statistical analysis revealed a statistically significant difference in the nephrotoxicity occurrence in patients with MDR-TB who had or not had impaired renal function before the TB treatment (80.0% and 21.4%, respectively; Chi-Square test, *p* = 0.017; Table 1). However, the logistic regression analysis and application of multivariate statistical tests revealed that none of the factors in this study had a significant effect on the nephrotoxicity occurrence (Supplementary Table S1). The test specificity was 91.2%, sensitivity was 61.5%, and 83.0% were correct predictions.

### 3.7. Full mtDNA Genome Sequencing Did Not Show Mitochondrial Association with ADR Occurrence

We used NGS-based full-length mtDNA sequencing to perform mtDNA haplogroup analysis and to reveal possibly unfavorable mtDNA variants. The results of the logistic regression analysis indicated that in our study, mtDNA haplogroups of patients with MDR-TB were not associated with the occurrence of either ototoxicity or nephrotoxicity (Supplementary Table S2). 

Furthermore, we assessed all detected mtDNA SNVs that were located in protein-coding genes; these SNVs could possibly lead to amino acid change or impact tRNA post-transcriptional modifications or rRNA. The results showed that all observed SNVs were sporadic, and no specific mutations, genes, or genome regions based on gene function could be clearly linked to either ototoxicity or nephrotoxicity events. The summary of this analysis is presented in Table 2 and Table 3.

Sequencing results also revealed that mtDNA mutations, which have been previously associated with aminoglycoside-induced ototoxicity, i.e., m.1555A > G [12], m.1494C > T [13], m.1095T > C [26], m.961T > G [27], m.961insC(n) [28], m.827A > G [29], and others [30,31], were not present in our cohort of patients with MDR-TB, whereas the m.961T > A variant, together with several other SNVs, was detected in one patient with ototoxicity (Table 3); however, the clinical significance of the detected variants remains unclear. Overall, the high heterogeneity and low frequencies of mtDNA SNVs were observed. This created a very variegated picture of SNV distribution that complicated the statistical analysis. Though many of the detected variants were missense and could be involved in post-transcriptional modifications, or tertiary folding, it was not possible to estimate the impact of those variants due to the low frequency and small sample size.

## 4. Discussion

We had several questions that we wanted to clarify by conducting this study. First of all, we aimed to specify the impact of selected factors on amikacin and capreomycin ADR—both ototoxicity and nephrotoxicity—in Latvian patients with MDR-TB. The ability of both antibiotic agents to cause side effects is well known; however, the penetrance of side effects could vary between different populations. To address this question, we inspected a wide range of different factors, including patient-, lifestyle-, and disease-related.

Among the different factors tested, the only statistically significant one for the ototoxicity appeared to be amikacin usage; patients with MDR-TB developed ototoxicity symptoms regardless of the age or the number of doses received. In a recent report, the results of the meta-analysis, including 18 studies from 10 countries around the world, showed that pooled prevalence of amikacin-induced ototoxicity was 38.93% (CI: 26.44–53.07%) in 545 patients with TB [32]. The reported prevalence of ototoxicity ranged between 7.0% and 75.0%, and our results on ototoxicity prevalence in patients with MDR-TB who received amikacin were close to the data described in studies in England in 2017 and 2013 (55% and 58%, respectively) [33,34]. It is currently accepted that ototoxicity occurrence is not dose dependent or treatment duration dependent, although there were some controversies in the scientific literature. In accordance with this opinion, our data did not show an ADR occurrence associated with the number of doses received. This finding emphasizes the role of patient variability and genetic background. For example, pre-existing or ongoing noise exposure affects the calcium channels and thereby could increase the aminoglycoside uptake in hair cells and accelerate its accumulation [6,35,36]. However, in our study, we did not observe any correlation between the ototoxicity events with previous hearing impairments. 

Our data indicated that nephrotoxicity was more frequently observed in older patients. This finding is in accordance with previous studies showing that patients who developed nephrotoxicity during aminoglycoside treatment had a higher average age [9]. Renal function impairment before the anti-TB treatment onset also appeared to be a nephrotoxicity-facilitating factor, although this finding was not confirmed by the multifactorial logistic regression analysis. In contrast, renal impairment before therapy was not associated with ototoxicity in our study; however, it was previously suggested that an altered renal function could impact the aminoglycoside clearance and accumulation in cochlear hair cells [37].

Our next question was about the possible effect of mtDNA genetic variables on the occurrence of ototoxicity and nephrotoxicity. Indeed, it was previously reported that ototoxicity manifestation can be influenced by patients’ age, nutrition and antioxidant deficiency, immunodeficiency, and also by mitochondrial functions and mtDNA variations [36]. Several ototoxicity-promoting mtDNA variants were previously reported in 12S rRNA-coding gene m.1555A > G [12], m.1494C > T [13], m.1095T > C [26], m.961T > G [27], m.961insC(n) [28], m.827A > G [29], and tRNASer (UCN) gene m.7444G > A and m.7445A > G [38].

In our study, a full mitochondrial genome analysis did not indicate any specific variants for the occurrence of adverse events, and none of the previously documented variants were present in our MDR-TB cohort. Thus, none of the ototoxicity events observed in our study could be linked to the previously reported ototoxicity-related mtDNA variants. 

Further, we looked deeper at the level of specific mtDNA variations to determine if they might be predisposition factors, as it is known that mitochondria are crucially necessary for cell metabolism, specific functions, and cell death regulation [39]. Overall, the observed variations, including those in the 12S rRNA and tRNA coding genes, showed high heterogeneity and low frequencies, as the majority of the detected variants were detected in single patients. Three possible ADR-related mtDNA variants were detected in the tRNA and rRNA genes: m.5654T > C (MT-TA), m.1709G > A (MT-RNR2), and m.961T > A (MT-RNR1). However, these SNVs were sporadic with unknown phenotypes and impact on mitochondrial metabolism, function, and/or ROS production. We can only speculate about the individual mutation impact on mitochondria functions and ototoxicity or nephrotoxicity development. However, the detected SNV m.961T > A variant, like the m.961T > G, could affect amikacin accumulation in cochlear hair cells [40]. This genetic locus attracted the interest of other studies looking for the hearing impairment-associated SNVs, and it was also examined in the heterologous inferential analysis (HIA) evaluating the possible disruptive potential of mtDNA rRNA mutations on ribosomal function [41]. The detected SNV m.961T > A variant was listed as undetermined regarding its impact on hearing loss, because the structural differences at the level of the mtDNA 12S rRNA secondary structure were too large to assign a heterologous equivalent and detect disruptive potential on ribosomal functions, which is the basis of the HIA method. In population studies carried out earlier, the variant m.961T > A has been reported twice: this variant was detected in one individual in our previous study of mtDNA mutation analysis in the ethnic Latvian population, as well as in one individual from Russia in the study performed by Dzhemilova and colleagues [42,43].

Considering other mitochondrial genetic aspects, it is observed that the mtDNA haplogroup can influence the phenotypic manifestation of hearing loss-associated mutations [44]. In our study, we did not find any associations between the occurrence of adverse events and mtDNA haplogroup; also, the distribution of macrohaplogroups did not differ from the general Latvian population [45]. However, it is also possible that the mtDNA haplogroup variability may cause heterogeneity of the results, variant expression, and penetrance, and there are discrepancies in the literature regarding mtDNA’s role in complex traits or diseases [41,46]. As one example, the mtDNA haplogroup could silence a possible pathogenic variant by providing a different range of expression [41,47]. Another aggravating factor is that the pathogenic effect of mtDNA mutations can be carried out through different mechanisms, and defects in mitochondrial translation machinery could be sensed and equilibrated by the cell itself [48,49].

Personalized medicine addresses the challenges for tailoring the right therapeutic strategy for the right person at the right time. However, currently available technology limits our ability to draw significant conclusions from any single case; also, interaction between genetic and non-genetic risk factors can often be hard to measure, and studies require a sufficiently large number of patients. Thus, in our study, we used a population-based approach and combined individual genetic and clinical data to assess the risks of ototoxicity and nephrotoxicity in TB patients, aiming to add novel information on the path to the personalization of TB treatment. On the other hand, because of sporadic occurrence, the detected mtDNA SNVs were recorded on an individual level, thus contributing to the creation of large global data sets that capture individual health trajectories.

Our study has several limitations. Our cohort size and the factor variability were the most significant limitations for the identification of all factors that may have an impact on ADR occurrence, as the individual variation is likely to obscure any factor–ADR relationship. The patient cohort was also collected in the period from 2014 to 2017, when injectable anti-TB medications were widely used in MDR-TB treatment. Nowadays, all oral regimens have been introduced according to the WHO guidelines on drug-resistant TB treatment; however, injectable agents such as amikacin can still be useful in severe cases of drug resistance [50]. In addition, amikacin is used to treat serious bacterial infections other than TB [5]. Thus, our findings could be useful on a broader scale.

## 5. Conclusions

In conclusion, we analyzed the occurrence of ototoxicity and nephrotoxicity in patients with MDR-TB receiving amikacin and/or capreomycin and evaluated the possible role of different patient-, disease-, and lifestyle-related factors. Ototoxicity development was more common in patients who received amikacin injections. No other factors showed a significant impact. Nephrotoxicity was likely associated with a previous renal health condition. Full mitochondrial genome sequencing results did not reveal any specific ADR-associated variants and showed no differences in adverse event occurrence for specific variants or the mtDNA haplogroup. The absence of the previously reported pathogenic mtDNA variants in our patients with ototoxicity and nephrotoxicity highlighted the complex nature of the ADR mechanisms. Although the mitochondrial role in aminoglycoside- and capreomycin-induced adverse events is widely discussed, further research is needed to gain a more detailed understanding of the drug–mitochondrial interplay.

## Figures and Tables

**Table 1:** The occurrence of ototoxicity and nephrotoxicity in patients with MDR-TB depending on various factors.

Characteristic		No. (%) of Patients	No. (%) of Patients with Adverse Events			
			Total	Ototoxicity *	Nephrotoxicity *	Both Events
Biological sex	Male	35 (74.5)	19 (54.3)	13 (37.1)	8 (22.9)	2 (5.7)
	Female	12 (25.5)	7 (58.3)	3 (25.0)	5 (41.7)	1 (8.3)
	*p* value **		1.000	0.505	0.269	1.000
Smoking status	Non-smoker	9 (19.1)	6 (66.7)	3 (33.3)	4 (44.4)	1 (11.1)
	Smoking experience	38 (80.9)	20 (52.6)	13 (34.2)	9 (23.7)	2 (5.3)
	*p* value		0.711	1.000	0.237	0.464
BMI	Underweight	8 (17.0)	5 (62.5)	3 (37.5)	3 (37.5)	1 (12.5)
	Normal	33 (70.2)	16 (48.5)	11 (33.3)	7 (21.2)	2 (6.1)
	Overweight	4 (8.5)	3 (75.0)	1 (25.0)	2 (50.0)	0
	Obese	2 (4.3)	2 (100.0)	1 (50.0)	1 (50.0)	0
	*p* value		0.387	0.936	0.463	0.822
Alcohol consumption	Increased	8 (17.0)	3 (37.5)	3 (37.5)	0	0
	Normal	39 (83.0)	23 (59.0)	13 (33.3)	13 (33.3)	3 (7.7)
	*p* value		0.437	1.000	0.086	1.000
HIV	Yes	5 (10.6)	3 (60.0)	2 (40.0)	2 (40.0)	1 (20.0)
	No	42 (89.4)	23 (54.8)	14 (33.3)	11 (26.2)	2 (4.8)
	*p* value		1.000	1.000	0.607	0.292
ART	Yes	3 (6.4)	2 (66.7)	1 (33.3)	1 (33.3)	0
	No	44 (93.6)	24 (54.5)	15 (34.1)	12 (27.3)	3 (6.8)
	*p* value		1.000	1.000	1.000	1.000
DM	Yes	6 (12.8)	4 (66.7)	3 (50.0)	2 (33.3)	1 (16.7)
	No	41 (87.2)	22 (53.7)	13 (31.7)	11 (26.8)	2 (4.9)
	*p* value		0.678	0.395	1.000	0.343
HCV	Yes	8 (17.0)	5 (62.5)	4 (50.0)	3 (37.5)	2 (25.0)
	No	39 (83.0)	21 (53.9)	12 (30.8)	10 (25.6)	1 (2.6)
	*p* value		0.716	0.416	0.666	0.071
TB incident case	New case	33 (70.2)	17 (51.5)	10 (30.3)	8 (24.2)	1 (3.0)
	Recurrent case	14 (29.8)	9 (64.3)	6 (42.9)	5 (35.7)	2 (14.3)
	*p* value		0.528	0.506	0.486	0.208
Injectable agent used	Amikacin	2	2 (100.0)	2 (100.0)	1 (50.0)	1 (50.0)
	Capreomycin	29	14 (48.3)	5 (17.2)	10 (34.5)	1 (3.4)
	Amikacin + capreomycin ***	16	10 (62.5)	9 (56.3)	2 (12.5)	1 (6.3)
	*p* value		0.282	**0.004**	0.222	**0.034**
Renal impairment before treatment	Yes	5 (10.6)	5 (100.0)	3 (60.0)	4 (80.0)	2 (40)
	No	42 (89.4)	21 (50.0)	13 (31.0)	9 (21.4)	1 (2.4)
	*p* value		0.056	0.320	**0.017**	**0.027**
Hearing impairment before treatment	Yes	31 (66.0)	16 (51.6)	10 (32.3)	8 (25.8)	2 (6.5)
	No	16 (34.0)	10 (62.5)	6 (37.5)	5 (31.3)	1 (6.25)
	*p* value		0.547	0.754	0.739	1.000
Treatment outcome	Cured	38 (80.9)	21 (55.3)	13 (34.2)	10 (26.3)	2 (5.3)
	Interrupted treatment	7 (14.9)	3 (42.9)	1 (14.3)	2 (28.6)	0
	Died	1 (2.1)	1 (100.0)	1 (100.0)	0	0
	Unknown	1 (2.1)	1 (100.0)	1 (100.0)	1 (100.0)	1 (100.0)
	*p* value		0.561	0.314	0.386	**0.002**
All patients		47 (100)	26 (55.3)	16 (34.0)	13 (27.7)	3 (6.4)

* Including the patients who experienced both adverse events. ** *p* values were calculated using a two-tailed Fisher’s exact test. Statistically significant *p* values are indicated in bold. *** Agents were used interchangeably, or therapy was changed based on the *M. tuberculosis* resistance data. HIV: human immunodeficiency virus infection; ART: antiretroviral therapy; HCV: hepatitis C virus infection; SNV: single-nucleotide variant; BMI: body mass index; DM: diabetes mellitus.

**Table 2:** The occurrence of ototoxicity and nephrotoxicity in patients with MDR-TB depending on mtDNA genetic variants.

Characteristic		No. (%) of Patients	No. (%) of Patients with Adverse Events			
			Total	Ototoxicity *	Nephrotoxicity *	Both Events
mtDNA haplogroup	H	19 (40.4)	11 (57.9)	7 (36.8)	6 (31.6)	2 (10.5)
	HV	1 (2.1)	0	0	0	0
	I	1 (2.1)	0	0	0	0
	J	3 (6.4)	2 (66.7)	0	2 (66.7)	0
	N	1 (2.1)	1 (100.0)	1 (100.0)	1 (100.0)	1 (100.0)
	T	6 (12.8)	4 (66.7)	4 (66.7)	0	0
	U	10 (21.3)	4 (40.0)	3 (30.0)	1 (10.0)	0
	V	3 (6.4)	2 (66.7)	0	2 (66.7)	0
	W	2 (4.3)	1 (50.0)	0	1 (50.0)	0
	X	1 (2.1)	1 (100.0)	1 (100.0)	0	0
SNVs in protein coding genes (only local private and global private SNVs)	yes	18 (38.3)	12 (66.7)	7 (38.9)	6 (33.3)	1 (5.6)
	no	29 (61.7)	14 (48.3)	9 (31.0)	7 (24.1)	2 (9.5)
	*p* value		0.245	0.753	0.521	1.00
SNVs in 12S rRNA gene (any)	yes	37 (78.7)	20 (54.1)	12 (32.4)	10 (27.0)	2 (5.4)
	no	10 (21.3)	6 (60.0)	4 (40.0)	3 (30.0)	1 (10.0)
	*p* value		1.000	0.716	1.000	0.521
SNVs in tRNA genes (any)	yes	26 (55.3)	15 (58.0)	11 (42.3)	7 (27.0)	3 (11.5)
	no	21 (44.7)	11 (52.4)	5 (23.8)	6 (28.6)	0
	*p* value		0.774	0.227	1.000	0.242
All patients		47	26 (55.3)	16 (34.0)	13 (27.7)	3 (6.4)

* Including the patients who experienced both adverse events. SNV: single-nucleotide variant. *p* values were calculated using a two-tailed Fisher’s exact test.

**Table 3:** mtDNA single-nucleotide variants detected in individuals with ototoxicity and nephrotoxicity, which were not associated with patients’ mtDNA haplogroups *.

Patient ID	mtDNA Haplo- Group **	Oto-Toxicity	Nephro-Toxicity	SNV	Gene	Gene Function	Effect of SNV	Association with Deafness or Hearing Loss [24]
MDR-A1	T2b8	+	+	m.9254A > G	MT-CO3	Protein coding	Synonymous	no
				m.15287T > C	MT-CYB	Protein coding	Missense	yes
				m.16213G > A	non-coding	non-coding	Unknown	no
MDR-A3	H1h1	+	−	m.14482C > T	MT-ND6	Protein coding	Synonymous	no
				m.146T > C	MT-ND6	non-coding	Unknown	no
				m.14564A > G	non-coding	Protein coding	Missense	no
MDR-A4	H3b + 16129	−	+	m.12879T > C	MT-ND5	Protein coding	Synonymous	no
MDR-A6 ***	H	+	−	m.3834G > C	MT-ND1	Protein coding	Synonymous	no
				m.12882C > T	MT-ND5	Protein coding	Synonymous	no
				m.73A > G	MT-ND5	non-coding	Unknown	no
				m.146T > C	non-coding	non-coding	Unknown	no
				m.13350A > G	non-coding	Protein coding	Synonymous	no
				m.16114C > T	non-coding	non-coding	Unknown	no
				m.16192C > T	non-coding	non-coding	Unknown	no
				m.16311T > C	non-coding	non-coding	Unknown	no
				**m.5654T > C**	MT-TA	tRNA	No change in three-dimensional interactions	no
				m.4395A > G	MT-TQ	tRNA	No change in three-dimensional interactions	no
MDR-B2 ***	H+152	+	−	m.15833C > T	MT-CYB	Protein coding	Synonymous	no
				m.3796A > G	MT-ND1	Protein coding	Missense	no
				m.73A > G	MT-ND4L	non-coding	Unknown	no
				m.10550A > G	non-coding	Protein coding	Synonymous	no
				m.16304T > C	non-coding	non-coding	Unknown	no
				m.16356T > C	non-coding	non-coding	Unknown	no
				m.16362T > C	non-coding	non-coding	Unknown	no
				m.5821G > A	MT-TC	tRNA	No change in three-dimensional interactions	yes
				m.4435A > G	MT-TM	tRNA	Involved in post-trans-criptional modifications	yes
				m.4336T > C	MT-TQ	tRNA	No change in three-dimensional interactions	yes
MDR-B4	V7a	−	+	m.195T > C	non-coding	non-coding	Unknown	no
MDR-B6	V	−	+	m.16189T > C	non-coding	non-coding	Unknown	no
MDR-B7	W1i	−	+	m.7080T > C	MT-CO1	Protein coding	Missense	no
				m.16179C > T	non-coding	non-coding	Unknown	no
MDR-C4	U3b2a1	−	+	m.1709G > A	MT-ND2	rRNA	Unknown	no
				m.5333T > C	MT-RNR2	Protein coding	Synonymous	no
MDR-C5	H1b1	+	−	m.3591G > A	MT-ND1	Protein coding	Synonymous	no
MDR-C7	H+195	−	+	m.310T > C	non-coding	non-coding	Unknown	no
MDR-D1	U5a1b1h	+	−	m.16093T > C	non-coding	non-coding	Unknown	no
MDR-D5	T1a1b	+	−	m.9438G > A	MT-CO3	Protein coding	Missense	no
				m.15323G > A	MT-CYB	Protein coding	Missense	no
MDR-D7	J1c4b	−	+	m.16093T > C	non-coding	non-coding	Unknown	no
MDR-E1	H11	+	−	m.8898C > T	MT-ATP6	Protein coding	Synonymous	no
				m.7389T > C	MT-CO1	Protein coding	Missense	no
				m.9554G > A	MT-CO3	Protein coding	Synonymous	no
				**m.4215A > G**	MT-ND1	Protein coding	Synonymous	no
				m.16278C > T	non-coding	non-coding	Unknown	no
				**m.961T > A**	MT-RNR1	rRNA	Unknown	no
MDR-E4	H5a1a	+	+	m.93A > G	non-coding	non-coding	Unknown	no
				m.16483G > A	non-coding	non-coding	Unknown	no
MDR-F4	U4a2	+	−	m.16145G > A	non-coding	non-coding	Unknown	no
MDR-F6	X2c1a	+	−	m.11662T > C	MT-ND4	Protein coding	Synonymous	no
MDR-G2	T2b4a	+	−	**m.6593A > G**	MT-CO1	Protein coding	Synonymous	no
MDR-G4	H1b2	−	+	m.4496C > T	MT-ND2	Protein coding	Synonymous	no
				m.11248A > G	MT-ND4	Protein coding	Synonymous	no
				m.12510C > T	MT-ND5	Protein coding	Synonymous	no
				m.14259G > A	MT-ND6	Protein coding	Missense	no
				m.73A > G	non-coding	non-coding	Unknown	no
MDR-G5	H17a	+	+	**m.8712C > T**	MT-ATP6	Protein coding	Synonymous	no
				m.152T > C	non-coding	non-coding	Unknown	no
				m.310T > C	non-coding	non-coding	Unknown	no
MDR-H4	H6c1	−	+	m.150C > T	non-coding	non-coding	Unknown	no
MDR-H6	J1c2t	−	+	m.9615T > C	MT-CO3	Protein coding	Synonymous	no
				**m.4892C > T**	MT-ND2	Protein coding	Synonymous	no
				m.13359G > A	MT-ND5	Protein coding	Synonymous	no
				m.310T > C	non-coding	non-coding	Unknown	no
				m.16209T > C	non-coding	non-coding	Unknown	no

+: side effect occurred, −: side effect didn’t occur. In bold–mtDNA global private SNV. * Hotspot variants not included. ** mtDNA haplogroup was assigned based on the sequencing results. *** Heteroplasmic SNVs were present.

## Data Availability

The data that support the findings of this study are available from the corresponding author upon reasonable request.

## Supplementary Materials

The following supporting information can be downloaded at: https://www.mdpi.com/article/10.3390/jpm13040599/s1, Supplementary Table S1: The results of logistic regression analysis for the significance of factors for ototoxicity and nephrotoxicity occurrence in patients with MDR-TB (N = 47); Supplementary Table S2: The results of logistic regression analysis for the significance of mtDNA-related genetic factors for ototoxicity and nephrotoxicity occurrence in patients with MDR-TB (N = 47).
