# Supplementary material for: Assessment of Amikacin- and Capreomycin-Related Adverse Drug Reactions in Patients with Multidrug-Resistant Tuberculosis and Exploring the Role of Genetic Factors
Source: J Pers Med. 2023 Mar 29;13(4):599. doi: 10.3390/jpm13040599 (PMC10145258; doi:10.3390/jpm13040599)
Supplement: Supplementary file 1 [file jpm-13-00599-s001.zip › jpm-2141583-supplementary.pdf]

Supplementary Table 1

The results of logistic regression analysis for significance of factors for ototoxicity occurrence in patients with MDR-TB (N=47). Statistically significant factors are highlighted in bold. *Ref* – reference.

Standardized coefficients for variable *Ototoxicity*

Source		Value	Standard error	Wald Chi-Square	Pr > Chi²	Wald Lower bound (95%)	Wald Upper bound (95%)
Age		0.062	0.246	0.064	0.801	-0.420	0.544
Sex							
	Female	*Ref*					
	Male	0.111	0.321	0.120	0.729	-0.517	0.740
Capreomycin injections							
	No	*Ref*					
	Yes	-0.228	0.251	0.828	0.363	-0.719	0.263
Amikacin injections							
	No	*Ref*					
	Yes	0.458	0.233	3.867	**0.049**	0.002	0.915
Previous renal impairment							
	No	*Ref*					
	Yes	0.181	0.242	0.558	0.455	-0.294	0.655
HIV infection							
	No	*Ref*					
	Yes	0.078	0.402	0.037	0.847	-0.709	0.865
ART							
	No	*Ref*					
	Yes	0.078	0.376	0.043	0.837	-0.659	0.814
HCV infection							
	No	*Ref*					
	Yes	-0.052	0.309	0.028	0.867	-0.657	0.554
DM							
	No	*Ref*					
	Yes	0.039	0.207	0.035	0.851	-0.367	0.445
BMI							
	Normal	*Ref*					
	Obese	0.092	0.206	0.198	0.656	-0.313	0.497
	Overweight	-0.002	0.205	0.000	0.992	-0.403	0.399
	Underweight	0.105	0.215	0.239	0.625	-0.316	0.525
Smoking experience							
	Non-smoker	*Ref*					
	Smoking experience	-0.150	0.326	0.210	0.646	-0.789	0.490
Alcohol use							
	Increased	*Ref*					
	Normal	-0.068	0.234	0.085	0.771	-0.528	0.391
MDR-TB occurrence							
	Primary TB	*Ref*					
	Recurrence of TB	0.188	0.201	0.873	0.350	-0.206	0.583

Supplementary Table 2

The results of logistic regression analysis for significance of factors for nephrotoxicity occurrence in patients with MDR-TB (N=47). *Ref* – reference.

Standardized coefficients for variable *Nephrotoxicity*

Source		Value	Standard error	Wald Chi-Square	Pr > Chi^2	Wald Lower bound (95%)	Wald Upper bound (95%)
Age		0.141	0.271	0.272	0.602	-0.389	0.671
Sex							
	Female	*Ref*					
	Male	0.164	0.289	0.324	0.569	-0.402	0.730
Capreomycin injections							
	No	*Ref*					
	Yes	-0.357	0.250	2.032	0.154	-0.847	0.134
Amikacin injections							
	No	*Ref*					
	Yes	-0.373	0.296	1.585	0.208	-0.953	0.208
Previous renal impairment							
	No	*Ref*					
	Yes	0.206	0.240	0.737	0.391	-0.265	0.677
HIV infection							
	No	*Ref*					
	Yes	0.009	0.466	0.000	0.984	-0.905	0.923
ART							
	No	*Ref*					
	Yes	-0.018	0.411	0.002	0.965	-0.824	0.788
HCV infection							
	No	*Ref*					
	Yes	0.279	0.340	0.673	0.412	-0.387	0.944
DM							
	No	*Ref*					
	Yes	0.065	0.265	0.060	0.807	-0.455	0.585
BMI							
	Normal	*Ref*					
	Obese	0.249	0.228	1.194	0.275	-0.197	0.695
	Overweight	0.168	0.202	0.696	0.404	-0.227	0.563
	Underweight	0.278	0.237	1.374	0.241	-0.187	0.742
Smoking experience							
	Non-smoker	*Ref*					
	Smoking experience	0.024	0.323	0.005	0.942	-0.609	0.657
Alcohol use							
	Increased	*Ref*					
	Normal	0.447	0.318	1.980	0.159	-0.176	1.071
MDR-TB occurrence							
	Primary TB	*Ref*					
	Recurrence of TB	0.089	0.205	0.189	0.664	-0.313	0.491
